# Results-Based Financing in Mozambique’s Central Medical Store: A Review After 1 Year

**DOI:** 10.9745/GHSP-D-15-00173

**Published:** 2016-03-25

**Authors:** Cary Spisak, Lindsay Morgan, Rena Eichler, James Rosen, Brian Serumaga, Angela Wang

**Affiliations:** a USAID | DELIVER PROJECT, John Snow, Inc., Washington, DC, USA; b Health Finance and Governance Project, Bethesda, MD, USA; c Health Finance and Governance Project, Broad Branch Associates, Washington, DC, USA; d USAID | DELIVER PROJECT, Avenir Health, Glastonbury, CT, USA

## Abstract

**Background:**

Public health commodity supply chains are typically weak in low-income countries, partly because they have many disparate yet interdependent functions and components. Approaches to strengthening supply chains in such settings have often fallen short—they address technical weaknesses, but not the incentives that motivate staff to perform better.

**Methods:**

We reviewed the first year of a results-based financing (RBF) program in Mozambique, which began in January 2013. The program aimed to improve the performance of the central medical store—*Central de Medicamentos e Artigos Medicos* (CMAM)—by realigning incentives. We completed in-depth interviews and focus group discussions with 33 key informants, including representatives from CMAM and donor agencies, and collected quantitative data on performance measures and use of funds.

**Implementation:**

The RBF agreement linked CMAM performance payments to quarterly results on 5 performance indicators related to supply planning, distribution planning, and warehouse management. RBF is predicated on the theory that a combination of carrot and stick—i.e., shared financial incentives, plus increased accountability for results—will spur changes in behavior. Important design elements: (1) indicators were measured against quarterly targets, and payments were made only for indicators that met those targets; (2) targets were set based on documented performance, at levels that could be reasonably attained, yet pushed for improvement; (3) payment was shared with and dependent on all staff, encouraging teamwork and collaboration; (4) results were validated by verifiable data sources; and (5) CMAM had discretion over how to use the funds.

**Findings:**

We found that CMAM’s performance continually improved over baseline and that CMAM achieved many of its performance targets, for example, timely submission of quarterly supply and distribution planning reports. Warehouse indicators, such as inventory management and order fulfillment, proved more challenging but were nonetheless positive. By linking payments to periodic verified results, and giving CMAM discretion over how to spend the funds, the RBF agreement motivated the workforce; focused attention on results; strengthened data collection; encouraged teamwork and innovation; and ultimately strengthened the central supply chain.

**Conclusion:**

Policy makers and program managers can use performance incentives to catalyze and leverage existing investments. To further strengthen the approach, such incentive programs can shift attention from quantity to quality indicators, improve verification processes, and aim to institutionalize the approach.

## Abstract (precis)

The RBF scheme, which paid incentives for verified results, steadily improved the CMS's performance over 1 year, particularly for supply and distribution planning. Key apparent success factors:

1) The CMS had full discretion over how to spend the funds
2) Payment was shared with and dependent on all staff, which encouraged teamwork.
3) Performance indicators were challenging yet achievable.
4) The quarterly payment cycle was frequent enough to be motivating.

Recommendations for future programs: focus on both quality and quantity indicators; strengthen results verification processes; and work toward institutionalizing the approach.

## BACKGROUND

In the decades following the end of a prolonged civil war, Mozambique made important strides in health, including a decrease in the under-5 mortality rate, from 219 per 1,000 live births in 1990 to 97 per 1,000 in 2011.^1^ However, serious gaps have persisted. The maternal mortality ratio remains high, at 480 deaths per 100,000 live births (2013)^2^; the prevalence of modern contraceptives is only 7% in rural areas (2011)^3^; and both HIV and malaria are hyperendemic—the national HIV prevalence is 10.6% (2014 estimates).^4^

One of the underlying challenges is the poor performance of the public health supply chain. The Central Medical Store—*Central de Medicamentos e Artigos Medicos* (CMAM)—is responsible for managing the procurement, importation, central-level warehousing, and distribution of medicines and commodities used by the public health system to provinces. Over the years, reductions to its administrative autonomy and to access to resources created operational challenges that hindered the organization’s ability to respond both to emergencies and to routine needs. An assessment of medicines procurement and the supply chain management system, undertaken in February 2011,^5^ found multiple shortcomings:

- Poor information accuracy and flow between the central, provincial, and district levels
- Ad hoc distribution of medicines from provinces to districts and health facilities
- Fragmented management responsibility
- Inflexible financing

Supply chains are foundational for any health system, encompassing “the planning and management of all activities involved in sourcing and procurement … and all logistics management activities. [This] also includes coordination and collaboration with … suppliers, intermediaries, third-party service providers, and customers.”^6^

For health supply chains to work—for the right goods to be received and delivered in the right quantities, in good condition, to the right place, at the right time, for the right cost—many actors working in different locations, with different responsibilities, need to be motivated to do their part. And they must be held accountable. These actors—from central-level planners and procurement specialists, to regional warehouse and transportation teams, to local storekeepers and service providers—depend on each other for timely and accurate information and a reliable supply of health commodities. One break in the chain, one delay, can have repercussions throughout the system, ultimately affecting whether families can access lifesaving medicines and commodities.

For health supply chains to work, many actors in different locations must do their part.

Public-sector supply chains in many low-income countries suffer from poor performance. A key contributor is the often misaligned incentives at work in highly centralized public-sector bureaucracies, where the responsibility for essential, but disparate, tasks are assigned to individuals who may have weak incentives to perform well or in concert with one another.^7^ Misaligned incentives may be found throughout the supply chain and may include low compensation for staff paid irrespective of performance; poor communication, coordination, and accountability arrangements; or insufficient resources for management to invest in the necessary infrastructure and other investments to support the long-term success.

Public-sector supply chains in many low-income countries suffer from poor performance, often due to misaligned incentives.

In Mozambique, underperformance at CMAM has negatively affected the functioning of the supply chain as a whole, resulting in inaccurate information about stock levels and expiries, as well as delayed and inefficient distribution. Many strategies have been tried to improve the performance of CMAM. A warehouse management system was introduced, for example, providing tools to better control and manage stock and data. A monitoring and evaluation (M&E) framework was developed and a dedicated M&E unit created within CMAM to routinely track performance. And an electronic payment system—e-SISTAFE—has enabled CMAM to pay some suppliers and manage limited funds, independent of the Ministry of Health’s Department of Administration and Finance (DAF).

Donor financial and technical support has been significant. CMAM receives technical assistance and commodities from the US Government (USG); operational funding and commodities from the World Bank; and commodities from the Global Fund to Fight AIDS, Tuberculosis and Malaria (The Global Fund). The USG alone invests an average of US$10–15 million annually for technical assistance to CMAM.

Additionally, the Ministry of Health and its partners have developed supportive action plans and policies, such as the Supply Chain Logistic Plan of Action 2012^8^ and the Pharmaceutical Logistics Strategic Plan 2013,^9^ which includes a performance indicator and monitoring framework. These plans identify several goals:

- Improved quality and timeliness of information flow between districts, provinces, and CMAM, and better use of this information for planning and procurement
- Better planning for distribution from provincial warehouses to the districts
- Stronger supervision and internal audit of province/district stores by CMAM

Despite these improvements, CMAM has lacked data to demonstrate improved supply chain outcomes, and stakeholders in Maputo believed that CMAM’s performance was not improving as expected.

In this context, result-based financing (RBF) was proposed, both to realign incentives and to catalyze other investments in CMAM. RBF refers to “any program that rewards the delivery of one or more outputs or outcomes by one or more incentives, financial or otherwise, upon verification that the agreed-upon result has actually been delivered.”^10^ RBF programs vary widely, but most address poor performance by providing performance payments for verified results and some measure of autonomy over how to spend the incentives.

Results-based financing (RBF) was introduced in Mozambique to improve the performance of the public health supply chain.

The vast majority of RBF programs in low- and middle-income countries provide incentives either to patients, to encourage and enable them to seek and access health services, or to health care providers, to increase the quantity and improve the quality of the services they provide. Where RBF is being tried, experience suggests it can have an impact on health and may strengthen the health system in the process.^12^

Although use of performance incentives is common in high-income country supply chains, until recently, few—if any—RBF programs directly targeted public health supply chains in low-income countries. Momentum is increasing, however, to develop RBF approaches that specifically target these health supply chains. By linking incentives with verified results, RBF is increasingly viewed as one way to motivate the supply chain workforce; focus attention on—and provide demonstrable evidence of—measurable results; strengthen data collection and information systems; and, ultimately, strengthen the supply chain and improve health.

This paper reports on how Mozambique used RBF to improve the performance of CMAM and the supply chain. RBF was incorporated into a fixed amount reimbursement agreement (FARA or FAR agreement), a type of assistance from the United States Agency for International Development (USAID) that disburses a fixed amount of funds based on outputs rather than inputs. FARAs closely align with RBF approaches: the agreements condition payments upon the achievement of specific, time-bound, target-based, verified results. Mozambique’s supply chain FARA was the first time RBF had been used in the public health supply chain to drive improvements in operational performance.

The agreement was designed in coordination with the Ministry of Health—*Ministerio da Saude,* or MISAU—and its key partners: USAID, the World Bank, and The Global Fund. All parties had an interest in leveraging past investments in and assistance to CMAM, as well as in creating synergies with planned and future initiatives. For example, a World Bank RBF project will aim to strengthen the supply chain at the provincial level, thus complementing USAID’s investments in strengthening central medical store performance.

The FARA was signed on December 6, 2012, between USAID and the Directorate of Planning and Coordination (DPC) within MISAU, with a 1-year performance period beginning January 2013. (The DPC coordinates MISAU’s national directorates. At the time of this review, CMAM was positioned under the National Directorate for Medical Assistance—*Direcção Nacional de Assistência Médica*, or DNAM. In April 2014, CMAM was promoted to the level of national directorate.)

## EVALUATION METHODS

To understand what drove performance improvements at CMAM under the FARA, in March 2014 (14 months after the performance period began), the authors—a representative from the USAID | DELIVER PROJECT and a representative from USAID’s Health Finance and Governance (HFG) project—completed in-depth interviews and focus group discussions with 33 key informants in Maputo. Using a semi-structured interview guide, the consultants met with program stakeholders, including representatives from CMAM, USAID and other donor agencies—the World Bank, UK’s Department for International Development (DFID), and the United Nations Population Fund (UNFPA)—as well as mid- and lower-level staff at CMAM.

Where possible, basic quantitative data points related to performance measures of supply and distribution planning and warehouse operations, as well as use of funds, were collected.

Results-based financing is predicated on the theory that financial incentives will spur changes in behavior. The authors hypothesized that performance incentives would improve supply chain performance through 3 principal pathways:

1. Improved staff motivation—leading to better attendance and job performance
2. Improved collaboration and cooperation
3. Increased investment in supply chain infrastructure

In analyzing data, we sought to understand the extent to which respondent narratives matched our hypotheses, the extent to which they differed, and whether they differed depending on the respondent’s position in the organization or the sectors in which they worked. The interview guide, as a flexible qualitative instrument, was modified as new themes were uncovered.

## THE INTERVENTION: RBF FOR THE PUBLIC HEALTH SUPPLY CHAIN IN MOZAMBIQUE

Most RBF agreements include several key elements:

- An incentive recipient, who stands to receive a financial incentive contingent upon verified results
- Performance indicators and targets that must be reached
- A process to verify results at a certain frequency (e.g., each quarter, twice a year, annually)
- The structure of the payment itself

### Incentive Recipient: CMAM—the Central Medical Store

The incentive recipient in the FARA was CMAM. The objective of the agreement was to improve the performance of the central medical store, and thereby spark improvements in the supply chain generally for all key commodities in Mozambique.

### Performance Indicators and Targets

The FARA specified 5 performance indicators (Table), which focused on 3 priority areas or *sectors*—supply planning, distribution planning, and warehouse operations. Each sector had a history of underperformance at CMAM and, if improved, could facilitate continuous improvements within the sectors as well as improvements in lower levels of the supply chain.

**TABLE:** Indicators, Targets, and Payment Amounts for the Mozambique Fixed Amount Reimbursement Agreement (FARA)

Functional Area	Milestones to Be Completed Each Quarter of Calendar Year 2013 *USAID will reimburse CMAM the value specified as USAID’s contribution, as noted below, upon certification by DPC and approval and acceptance as certified by USAID of the following milestones at the end of each quarter of calendar year 2013.* a	USAID’s Contribution (Fixed Reimbursement Amount) per Quarter
Supply planning/ forecasting	(a) Annual quantification plan that meets 3 predetermined criteria, for each product group,^b^ *or* (b) Quarterly updated supply plan that meets 3 predetermined criteria, for each product group^b^	$25,000
Distribution planning	Order cycle time for distribution planning is 15 calendar days or less for via Clássica orders to the 18 central + provincial clients	$25,000
Warehouse	Order pick/pack accuracy for distribution to the 18 central + provincial clients is 84% for Q1, 86% for Q2, 88% for Q3, and 90% for Q4	$25,000
Warehouse	Order cycle time for dispatch is 35 calendar days or less for via Clássica orders to the 18 central + provincial clients	$25,000
Warehouse	Inventory accuracy in central warehouses that have implemented the MACS warehouse management system is 75% for Q1, 78% for Q2, 81% for Q3, and 84% for Q4	$25,000
**Quarterly Total**		**$125,000**
**Total for Calendar Year 2013**		**$500,000**

Abbreviations: CMAM, *Central de Medicamentos e Artigos Medicos* (Mozambique's central medical store); DPC, Directorate of Planning and Coordination; USAID, United Sates Agency for International Development.

a Indicators were weighted equally. Scores for the warehouse indicators were taken in aggregate from all 3 warehouses, with targets derived from aggregate warehouse performance.

b Product groups: HIV/AIDS, malaria, tuberculosis, essential medicines, laboratory, reproductive health, vaccines, and medical materials.

The **supply planning** sector used planning and quantification reports to inform annual procurement and funding decisions, as well as periodic inventory replenishment activities. Each quarter, the Medicines Working Group—*Grupo de Trabalho de Medicamentos* (GTM)—convened meetings for each of its product category “subgroups” to agree on quarterly commodity supply plans or annual quantification plan reports. The supply planning performance indicator required these quarterly reports to be submitted on time, and according to specific quality criteria. Eight product category subgroups were measured by this indicator: HIV/AIDS, malaria, tuberculosis, essential medicines, laboratory, reproductive health, vaccines, and medical materials.

Similarly, **distribution planning** is an important step in CMAM’s order fulfillment cycle—when orders are validated and inventory allocated, and if necessary, rationed. Timely distribution planning could enable more timely delivery of orders to provincial and district levels, thereby reducing the risk of stock-outs at lower levels.

**Warehouse** indicators measured the accuracy of filling quarterly orders (“order pick and pack” accuracy); the time required to dispatch quarterly orders to recipients; and the ongoing maintenance of accurate physical inventory against inventory records.

The indicators chosen for the FARA were considered *core* supply chain performance indicators: areas on which broader supply chain performance improvements would rely. For example, measuring and improving inventory accuracy is necessary *before* other inventory- and finance-related data—such as product leakage or expiry rates—can be measured.

Only indicators that could be routinely measured and verified were chosen, and data sources and reporting mechanisms were validated or created for each indicator. Additionally, all of the indicators were defined with targets to be measured on a quarterly basis. This ensured that performance data were being collected and monitored on a regular basis, facilitating periodic evaluation and adjustment, and that staff were continuously working toward a near-term goal. This structure also aligned with the common commercial-sector practice of quarterly reporting of business results.

The targets themselves were set based on baseline performance and at levels that presented a reasonable challenge while nonetheless being within CMAM’s control to achieve. In other words, the targets were not too easy, nor too hard. For 2 indicators, “order pick and pack accuracy” and “inventory accuracy,” the quarterly targets became increasingly more difficult each subsequent quarter.

Performance targets were not too easy nor too hard to achieve.

CMAM staff felt strongly that each performance indicator should measure the performance of an individual sector, rather than multiple sectors; staff members did not want their own results to be affected by the performance of others. This is in keeping with a lesson learned in many other RBF programs: that staff members should only be held accountable for (and thus rewarded or penalized for) something that is in their control to achieve. Otherwise, RBF may have the opposite of the intended affect: it may demotivate instead of motivate.

### Verification

Verification is an essential component of RBF, validating that reported results meet the targets, time lines and other agreed-upon criteria. A robust verification process helps manage risk for all parties in an RBF agreement. Each quarter, after CMAM submitted results, a team at USAID verified those results. The team initially consisted of 2 supply chain experts and was expanded later to include 2 members of USAID’s M&E team. The verification team used a standardized checklist to ensure the integrity of the verification process.

### Structure of Incentive Payment

In most RBF programs, the potential incentive payment is determined, first by considering preexisting incentives (salaries, per diems, etc.), and then by considering the level of effort required to motivate improved effort, creativity, teamwork, and, ultimately, performance. The incentive payment is not intended to reimburse, for example, a health facility for the costs associated with increasing the number of immunizations it conducts. Rather, the incentive is an *additional* payment, calibrated to be high enough to be motivating, but not so high that it introduces perverse incentives (by, for example, focusing attention away from non-incentivized tasks).

The incentive payment should be high enough to be motivating but not too high that it introduces unintended negative consequences.

The mode for determining the performance payment differed somewhat from what is normally done in RBF programs. Because the FARA is a *reimbursement* financing mechanism, performance payments are normally determined by estimating the costs associated with completing activities and achieving targets. In the case of Mozambique, USAID considered unfunded items in CMAM’s annual budget; identified those that might enable CMAM to improve its performance on the 5 performance indictors; and used the total of these items to create a budget for the performance payment (or “reimbursement” in USAID parlance). So as not to incentivize efforts related to one indicator over another, the potential reimbursement was split evenly across the indicators; that is, each of the 5 indicators was “worth” one-fifth (20%) of the total possible payment. The Table summarizes the set of indicators, the targets, the total cost of the activities, and the agreed-to payment or reimbursement schedule.

**FIGURE 1.**
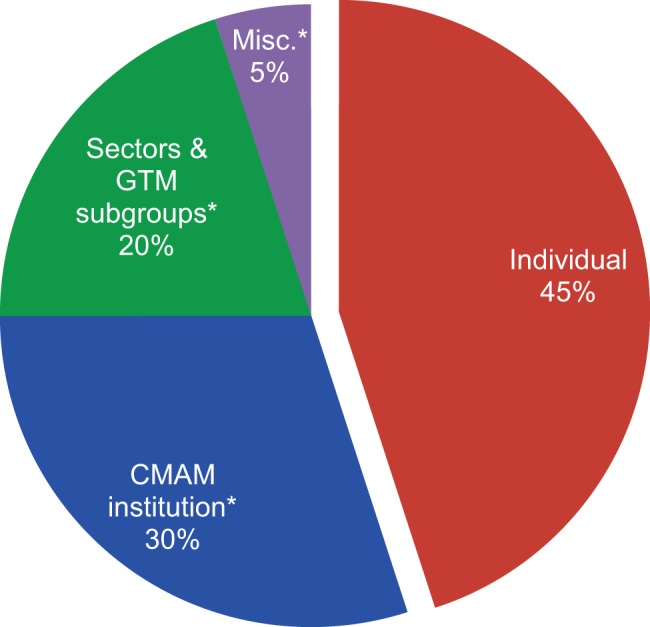
CMAM Allocation of Received Funds From Results-Based Financing Program Abbreviations: CMAM, *Central de Medicamentos e Artigos Medicos* (Mozambique’s central medical store); GTM, *Grupo de Trabalho de Medicamentos* (Medicines Working Group). * Split of institutional allocation between CMAM general, sectors and GTM subgroups, and miscellaneous is approximate.

The FARA gave CMAM complete discretion in its use of the performance payment and did not require CMAM to provide proof of how it used the funds. In this way, the Mozambique RBF program is not unlike RBF programs targeted at health care providers, many of which allow health workers control and discretion over how to spend funds and consider such autonomy a key motivating factor in RBF. CMAM proposed a plan for allocating the funds, which was approved by the Minister of Health: 55% were invested in the institution, with portions of this reserved for the sectors responsible for the indicators and for the GTM product category subgroups, which, as noted earlier, contribute to the planning indicator (Figure 1). The remaining, and still substantial, share of funds (45%) was shared among all staff as personal incentives, based on an established formula that considered the rank and category of staff.

## FINDINGS

During the first year of the agreement, steady progress was made on achieving the performance targets, particularly for planning and distribution. Progress was not as steady, but was nonetheless positive, for the warehouse performance targets. At the time of this review, data for the fourth quarter of the first year were not yet available for all indicators.

### Supply Planning

To meet the target for the supply planning indicator, each of the 8 GTM product category subgroups submitted in a timely manner a quarterly planning report—either the annual quantification report or a supply plan update—that met specified quality criteria, such as documented participation by named key stakeholders.

**FIGURE 2.**
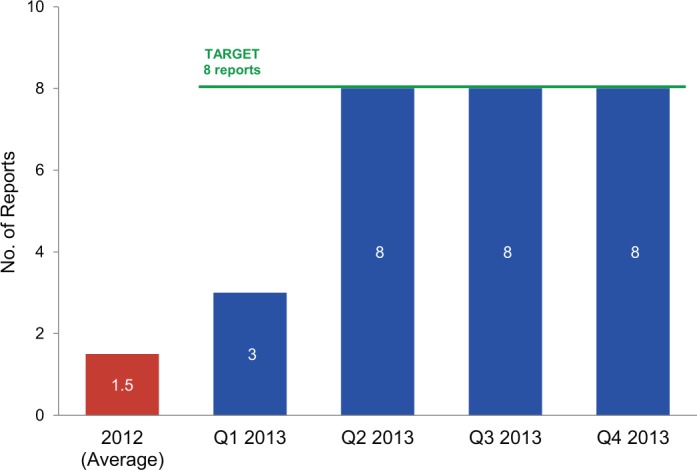
Supply Planning Results: Number of Supply Chain Reports Meeting Timely Submission Before (2012) and 1 Year After the Results-Based Financing Agreement Was Initiated in January 2013

CMAM’s baseline performance for the supply planning indicator was between 1 and 2 reports submitted each quarter (Figure 2), which, informants noted, were rarely completed in a timely or participatory manner. However, after a misstep in the first quarter, when only 3 of 8 reports were submitted and verified, the planning sector consistently achieved its targets for this indicator.

### Distribution Planning

**FIGURE 3.**
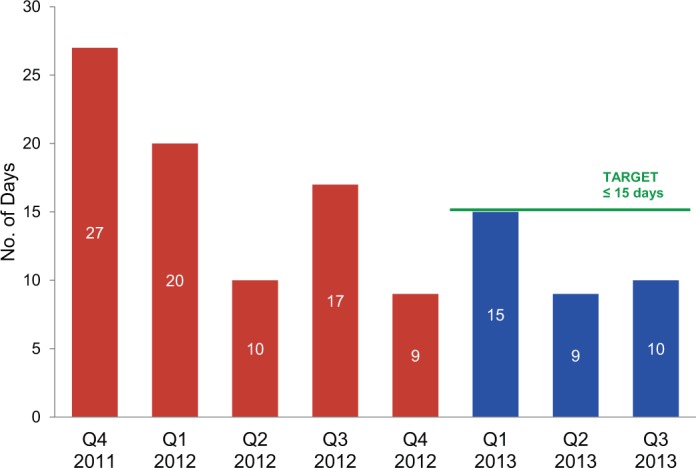
Distribution Planning Results: Number of Days From Receipt of Orders to Completion of Distribution Plan Before (2011–2012) and 1 Year After the Results-Based Financing Agreement Was Initiated in January 2013^a^ ^a^Results for the fourth quarter of 2013 were not yet available at the time of this review.

To meet the target for the distribution planning indicator, the distribution sector had to develop and submit a quarterly distribution plan within 15 days of the beginning of the planning cycle. Past performance on this indicator was inconsistent; baseline performance for the year prior (2012) and the last quarter of 2011 was as low as 9 days and as high as 27. However, the sector consistently submitted the distribution plan within the 15-day target during the first year of the RBF agreement (Figure 3).

### Warehouse

**FIGURE 4.**
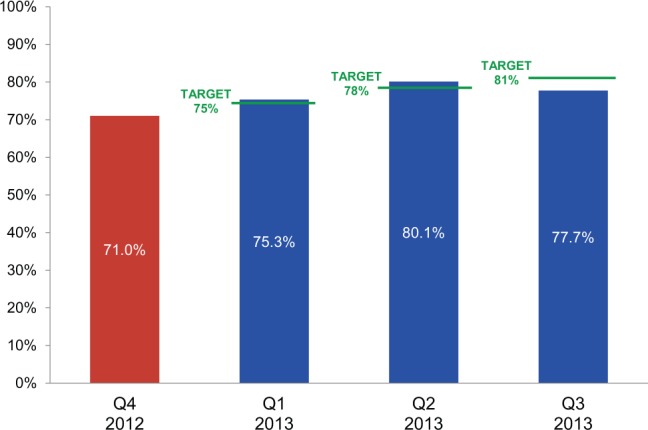
Inventory Accuracy Results: Percentage of Electronic Inventory Records Matching Physical Inventory Counts Before (Quarter 4 in 2012) and 1 Year After the Results-Based Financing Agreement Was Initiated in January 2013 ^a^ ^a^ Results for the fourth quarter of 2013 were not yet available at the time of this review. Target for the fourth quarter was set at 84%.

Warehouse indicators, which monitored inventory management, order fulfillment, and delivery, proved more challenging. To meet the inventory accuracy indicator, a physical inventory count had to match an electronic inventory report within 1 percent margin of error, for a specific number of unique items (stock keeping units or SKUs) selected by random sampling. Maintaining accurate physical inventory and inventory records is critical to ensuring availability of commodities to fill orders and to good use of limited resources. The target for inventory accuracy was set based on historical performance and was increased incrementally each quarter throughout the year. CMAM achieved the inventory accuracy target during the first 2 quarters, but not in the third quarter. Nonetheless, measurable improvement in inventory accuracy was seen throughout the year (Figure 4).

**FIGURE 5.**
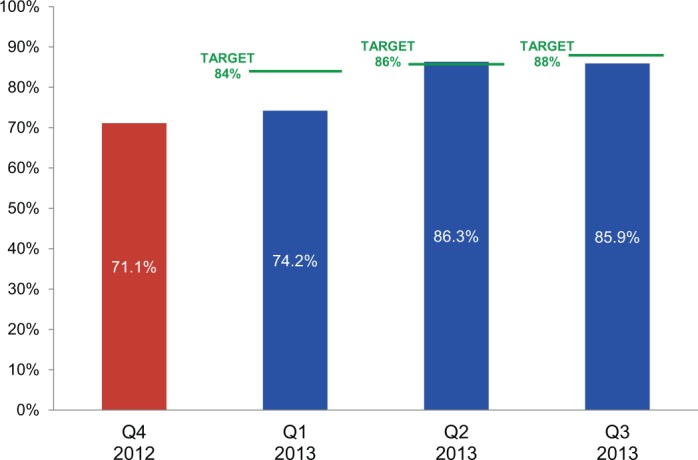
Order Pick and Pack Accuracy Results: Percentage of Products Shipped Matching Packing List in Distribution Plan Before (Quarter 4 in 2012) and 1 Year After the Results-Based Financing Agreement Was Initiated in January 2013 ^a^ ^a^Results for the fourth quarter of 2013 were not yet available at the time of this review. Target for the fourth quarter was set at 90%.

The distribution plan drives the order fulfillment process, which is executed in the warehouse. The order pick and pack accuracy indicator was met when the number and quantity of products that were shipped to the customer matched the number and quantity approved to be shipped in the distribution plan. The target for this indicator was increased incrementally each quarter. This target was achieved only in the second quarter; however, performance continued to improve throughout the year (Figure 5). Fourth-quarter data were not available at the time of the assessment.

**FIGURE 6.**
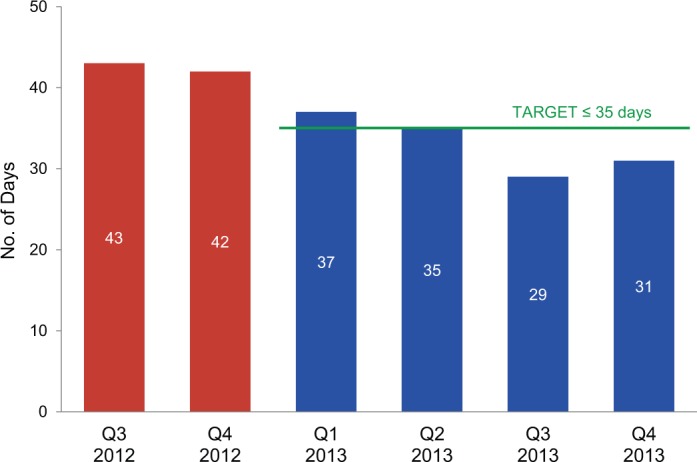
Dispatch Cycle Time: Number of Days From Receipt of Distribution Plan to Delivery of Health Commodities Before (Quarter 3–4 in 2012) and 1 Year After the Results-Based Financing Agreement Was Initiated in January 2013

The dispatch cycle time indicator measured the timeliness of delivering orders to clients. This indicator was achieved when quarterly shipments were received by clients within 35 calendar days after the warehouse received the distribution plan. Baseline performance for this activity was around 42 days. Although the target was not met in the first quarter, it was met or exceeded in subsequent quarters (Figure 6).

Difficulty in meeting the warehouse targets was due, in part, to such context-specific issues as out-of-service equipment; low staff levels; and Internet connectivity problems, which sometimes made it difficult to connect to the warehouse management system. Some problems may be resolved in the future if CMAM uses incentive funds to maintain equipment, but others, such as lack of staff or high staff turnover, require longer-term solutions that CMAM may not be able to control.

Warehouse performance indicators proved more challenging to meet than supply and distribution planning indicators.

It is also important to note that performance improved for all warehouse indicators, *even during quarters when targets were not met*. This is because the warehouses were rewarded on the basis of their aggregate scores on indicators, masking variances among them. For example, in relation to the order pick and pack accuracy indicator, the target for the third quarter of 2013 was 88%, whereas the aggregate score was 85.9%, meaning this indicator was not met for that quarter. However, a look at their individual scores shows a large variance:

- Zimpeto: 94.4%
- Beira: 65.8%
- Adil: 47.8%

The existing conditions at the warehouses seem to have a strong link to the individual warehouse results. Zimpeto central warehouse is the largest and best-equipped warehouse and is owned by CMAM. Located about 45 minutes outside downtown Maputo, it is well-equipped, well-organized, well-staffed, and well-lit. Zimpeto is, not unexpectedly, the best performer among the warehouses. The Adil warehouse is an hour outside Maputo in the opposite direction. Adil is a rented facility that is only partially equipped with racks, poorly lit and ventilated, and understaffed. Beira warehouse, an hour’s flight north of Maputo, is similar in condition to Adil; it does not have racking or other features and, according to CMAM and its technical assistance partners, is generally the lowest performing warehouse of the three.

## WHAT ABOUT RBF DROVE PERFORMANCE IMPROVEMENTS?

What about RBF spurred the improvements described above? In addition to the incentive itself, several characteristics of the performance payments appear to have motivated CMAM staff to exert extra effort and improve their performance.

### Significance of Incentive Payment for Individuals

Although the total possible FARA payment was only about 5% of the total operational investment and support CMAM receives, for individuals at CMAM, the FARA payment was significant relative to the overall compensation package of CMAM staff. In addition to their salaries, each CMAM staff member received an individual bonus each month, as well as per diem and allowances for travel. The monthly bonus had been in place since 2011, and its allocation was based on a formula that considered each staff member’s job description and rank. Eligibility for the bonus depended on individual performance and attendance. FARA payments were allocated among staff based on this established formula. Although the quarterly FARA payments were smaller than the monthly bonuses, a review against the salary scale suggested that, if CMAM achieved the targets for all indicators in 1 quarter, the amount paid to each staff member for RBF in that quarter could represent as much as 1 month’s salary.

### CMAM Discretion Over How to Spend Funds

CMAM had full discretion over how to spend the RBF incentive payment, a crucial difference from other financing sources, and it was not required to report or reconcile back to USAID on use of the funds. Discretion and autonomy were motivating: they allowed CMAM to address small, ad hoc needs, such as purchasing distilled water for the forklift batteries or packing materials, thereby reducing CMAM’s dependence on partners and providing CMAM the ability to show fiscal responsibility.

CMAM had full discretion over how to spend the RBF payment, a key factor in motivating better performance.

Moreover, CMAM management employed a democratic process with sector heads to agree on how the institutional portion would be spent. At the end of each quarter, CMAM management solicited a list of priority items and estimated cost from each department. Together, the *colectivo* (i.e., the senior management team representing each department within CMAM) decided how to spend the institutional portion.

Interestingly, although the FARA did not require CMAM to report the use of funds to USAID, after the first tranche was paid, CMAM voluntarily shared the details of how it spent the funds with USAID. After CMAM received its first incentive payment, it was able to invest in infrastructure improvements; purchase of office equipment, supplies, and materials; travel costs; and workforce environment improvements such as curtains and chairs for shared spaces.

Staff members repeatedly praised these investments and small improvements as contributing to their workplace satisfaction and morale; they brought a sense of professional pride. Moreover, sector heads said they valued having a voice in spending decisions and seeing tangible results. The participatory process also contributed to a greater sense of transparency within the organization.

### Incentive Payment Was Shared With Everyone—and Depended on Everyone

Unlike the existing monthly bonus scheme mentioned above, which is based on each individual’s performance, the RBF payment was based on *team* performance. Although only a few sectors were *directly* responsible for achieving targets and reporting on the indicators, it was widely recognized that all staff had supporting roles, even if they were indirect. Interdependency to get things done was not new at CMAM, but its importance was amplified because of the FARA, where group performance translated into a tangible, personal benefit. The prospect of personal and collective gain, along with increased accountability and scrutiny of their work, seem to have created a virtuous cycle among CMAM staff members that encouraged extra effort, better attention to detail, teamwork, and collaboration.

Staff members repeatedly said they were working harder, longer hours to make sure tasks and reports were completed, adhering to processes, and using existing tools. Warehouse staff repeatedly spoke of paying more attention to detail and following standard operating procedures more closely. As an example, staff at Adil described how they began double-checking order packing accuracy: one person picked the product and another confirmed it was the correct product and quantity, even though dedicated staff members were not available for this purpose. Although a number of tools and reports were already available to CMAM staff, the data collection, measurement, and supporting documentation that were required to report performance results for the FARA encouraged their regular use.

Staff also recounted working together more closely—increasing collaboration and coordination within and among teams—and they described being proactive in seeking out data that were not forthcoming. This is important because meeting the planning and distribution indicator targets depended on the cooperation of actors from outside the responsible sectors. The head of planning at CMAM noted that, because of RBF, he was able to demand regular and timely reports from the internal technical subgroups. In the past, the groups did not regularly submit reports.

Similarly, to prepare the distribution plan before its deadline, the distribution sector relied on timely order submission from the provinces. The distribution sector head noted that during the FARA she notified provinces that if their requisitions were late, they would have lower priority during the distribution planning process. In this way, the promise of a reward spurred the distribution sector’s creativity in problem solving and ability to influence changes outside the department.

### Prospect of Losing the Incentive

Interestingly, the prospect of losing money also seems to have spurred improvements in performance. Although staff members had an opportunity to earn “money in our pockets,” as it was described, the impact of the FARA on workplace norms was not immediate. It took the first quarter’s failures and near misses to illustrate both what the staff stood to gain personally and what was possible for CMAM to receive as a whole. At the end of the first quarter, CMAM met only 2 of 5 indicators and received only 40% of the possible incentive payment (under the FARA, CMAM received zero payment for unmet targets). In the second quarter, with a better grasp of the program and what was at stake, CMAM achieved all the targets.

## CHALLENGES

As with any RBF approach, unintended consequences are possible; elements meant to motivate can sometimes have the opposite effect. It is possible, for example, that jointly rewarding the warehouses had a demotivating effect. In the initial design, the high-performing warehouse(s) were, in essence, penalized by low performers. Over time, this could demotivate staff. It may also drive unintended distortions, such as gaming of the program—in other words, manipulating results for better outcomes. To address potential perverse incentives, the program may consider configuring the warehouse indicators differently, such as giving each warehouse specific targets against their own baseline.

There also appears to be some evidence that the program’s approach to sharing the reward among all staff, regardless of individual contribution to achieving the targets, may have caused frustration. Any program where incentives are shared can potentially face the free-rider problem. Some people will inevitably work, or feel they are working, harder than others. Indeed, some CMAM staff members noted frustration that they worked overtime to achieve targets, while others in their department did not, and yet the incentive did not vary according to level of effort. This design element was deliberate: it intended to drive increased accountability within CMAM, and this did appear to be happening, as discussed previously. However, this is an area that should be monitored to minimize any negative impact on morale. In the future, it may make sense to consider including an individual performance component in the allocation formula.

## KEY LESSONS

Despite challenges, overall, Mozambique’s experiment with RBF for the supply chain resulted in tangible and measurable positive change. Below are key lessons related to design, implementation, and future attempts at incorporating performance incentives in public health supply chains.

### Program Design

In many health systems areas, funding and know-how are not enough to spur performance improvements. RBF is intended to address the “black box” of motivation—the gap between necessary funding, inputs and training, and actual effort. As such, the goal is behavior change: increased motivation and effort. For this reason, the design of any RBF program must place motivation at the center of design decisions, which Mozambique’s FARA achieved.

Among our key design lessons are:

#### Careful and deliberative design

Stakeholders involved in Mozambique’s FAR agreement allowed the necessary time (nearly 1 year) to design the agreement, ensuring that all the stakeholders understood who was accountable for what. RBF will not motivate if stakeholders do not understand what they are being held accountable for.

#### Collaboration

Buy-in is a necessary precondition for increased motivation. The FARA design was a collaboration between USAID and CMAM, which ensured indicators were not imposed, but agreed-to as a group. The design was an iterative, consultative process with CMAM, Health Systems 20/20 (the USAID project that preceded HFG), USAID’s Supply Chain Management System (SCMS), USAID, the USAID | DELIVER PROJECT, and the World Bank, in which all participants debated and came to consensus on both the indicators and the implementation.

#### Specificity

The FARA clearly described each element of the agreement, including deadlines, precise indicator definitions and targets, and data sources for verification and reporting mechanisms. This ensured that the expectations and requirements were clearly defined and agreed to.

#### Achievable challenge

Performance indicators were the right mix of being challenging without being too difficult, and, conversely, were within CMAM’s reach to achieve without being too easy. Moreover, they were relevant metrics, based on standard supply chain performance indicators; they were rigorously defined and tested during the design process.

#### Frequency of payment cycle

The quarterly payment cycle was frequent enough to be motivating and helped create routines among CMAM staff. Teams had renewed opportunities to meet their targets in spite of earlier failures, and quarterly deadlines were too close for staff to lose sight of them. Staff were constantly working toward *new* near-term deadlines and targets; quarterly payments kept the goal well within sight and reach and continuously offered the opportunity to achieve the desired results or lose the benefit.

The quarterly payment cycle was frequent enough to be motivating.

### Implementation

The design of a program is only a template—a theory. Impact depends on how innovations are implemented. For this, the RBF FARA was also strong, in 2 important ways.

#### Openness to learning and revision

RBF programs are not and should not be considered static. The flexibility to evolve as lessons were learned was an important element of the positive results of the FARA in Mozambique. For example, after testing data collection and reporting for the stock accuracy indicator in late 2012, USAID agreed to change the precise definition of the stock accuracy indicator to allow for a 1 percent margin of error in stock accuracy. Without this revision, the indicator would have been unachievable. Openness to learning and revision resulted in a relevant and achievable indicator.

RBF programs should evolve as lessons are learned.

The process for verification also evolved—going from a 2-person supply chain team to a larger team that included both supply chain and M&E experts. As the verification process was strengthened, the time from the submission by CMAM of quarterly results to the time when USAID disbursed funds was reduced from 50 days to about 30 days.

For future iterations of the agreement, stakeholders should revise the design—indicators, targets, verification—to reflect new and changing realities, while at the same time ensuring that the program remains challenging. Maintaining some core elements or core indicators may be appropriate and helpful in promoting the institutionalization of priority functions or processes, reinforcing routines and best practices.

#### Effective sensitization by CMAM

Effective sensitization is a foundation for any RBF program: people will not be motivated by a program they do not understand. Prior to the start of the performance period, CMAM management made sector heads responsible for communicating the program and expectations to their respective teams. No formal communication plan was set up, but the key points appeared to have been well disseminated throughout the organization, including the setting of specific targets and deadlines, that benefits would be paid only for targets that were met, and that all staff would share the benefits of meeting the targets.

## RECOMMENDATIONS FOR STRENGTHENING THE APPROACH

### Strengthen incentives for quality

Many RBF programs in service delivery have evolved from rewarding health care providers for increases in the *quantity* of services delivered to improvements in the *quality* of care. RBF approaches targeted at the supply chain may see a similar trajectory. For example, in Mozambique, the quantification and supply plan indicator initially focused on the *timeliness* of reports; efforts then shifted to focusing on their *quality*.

Just as RBF programs in service delivery have shifted from a focus on *quantity* of services delivered to improvements in *quality* of care, so too can supply chain-RBF programs evolve.

In future iterations, incentives for quality can be strengthened. The program may consider, for example, more closely evaluating reports against quality criteria; providing feedback when a report fails to meet the criteria; and building a culture of quality improvement and data use more generally. At CMAM, informants at the warehouses did not know either their individual warehouse performance scores or their contribution to the overall warehouse results. Access to performance data and sharing results within CMAM may further support morale and performance improvements.

### Strengthen M&E and verification to detect distortions

Verification and M&E are essential components of any RBF program, both as the means for detecting gaming and other distortions and for ensuring that the results that are paid for are real. USAID refined its approach to verification, but it could be further clarified and strengthened. As in other RBF programs, it is necessary to develop a verification process that is rigorous without being prohibitively expensive or burdensome.

In addition, CMAM’s capacity to monitor performance data should be strengthened, in part to ensure that non-incentivized indicators are not being neglected. At the time of this review, CMAM’s M&E department was still relatively new and was responsible for monitoring 26 performance indicators in its M&E plan (which includes the RBF indicators). While no evidence suggests neglect of the other indicators in Mozambique, stakeholders expressed concern about the potential for this in the future.

### Aim to institutionalize the approach

Over the long term, governments, such as the Government of Mozambique, may consider institutionalizing the RBF approach—that is, rewarding verified results—both at the central level and throughout the supply chain. As the payment amount in this intervention is modest relative to CMAM’s overall budget, it is conceivable to imagine the government assuming responsibility for funding in the future. However, capacity is required for refining the systems for updating indicators, setting targets, and, critically, verifying performance. Over time, with a more fully funded operational budget, it may be possible for continued performance improvements to offset the cost of maintaining such an intervention.

## Notes

**Peer Reviewed**

**First published online:** 2016 Mar 2
